# Quantifying Cytosolic Cytochrome c Concentration Using Carbon Quantum Dots as a Powerful Method for Apoptosis Detection

**DOI:** 10.3390/pharmaceutics13101556

**Published:** 2021-09-25

**Authors:** Cristian Silviu Moldovan, Anca Onaciu, Valentin Toma, Radu Marginean, Alin Moldovan, Adrian Bogdan Tigu, Gabriela Fabiola Stiufiuc, Constantin Mihai Lucaciu, Rares Ionut Stiufiuc

**Affiliations:** 1 Department of Pharmaceutical Physics & Biophysics, Faculty of Pharmacy, “Iuliu Hatieganu” University of Medicine and Pharmacy, Louis Pasteur Street No. 4-6, 400349 Cluj-Napoca, Romania; moldovan.cristian@umfcluj.ro (C.S.M.); anca.onaciu@umfcluj.ro (A.O.); clucaciu@umfcluj.ro (C.M.L.); 2 Medfuture-Research Center for Advanced Medicine, “Iuliu Hatieganu” University of Medicine and Pharmacy, Marinescu 23/Louis Pasteur Street No. 4-6, 400337 Cluj-Napoca, Romania; valentin.toma@umfcluj.ro (V.T.); margi.radu@outlook.com (R.M.); alin.moldovan92a@gmail.com (A.M.); bogdan.tigu@umfcluj.ro (A.B.T.); 3 Faculty of Physics, “Babes Bolyai” University, Mihail Kogalniceanu Street No. 1, 400084 Cluj-Napoca, Romania; gabriela.stiufiuc@ubbcluj.ro

**Keywords:** carbon dots, fluorescence, cytochrome c, apoptosis, cells

## Abstract

Background: Cytochrome c (Cyt c) is a key biomarker for early apoptosis, and many methods were designed to detect its release from mitochondria. For a proper evaluation of these programed cell death mechanisms, fluorescent nanoparticles are excellent candidates due to their valuable optical properties. Among all classes of nanoparticles developed thus far, carbon-based quantum dots bring qualitative and efficient imaging strategies for biomedical applications as a consequence of their biocompatibility and low cytotoxicity. Methods: In this study, we synthesized carbon quantum dots smaller than 5 nm from sodium citrate and polyethylene imine. These nanoparticles were rigorously characterized, and their quenching capacity in apoptotic events was assessed in A549 cells treated with staurosporine and etoposide. For the evaluation of Cyt c release, a phenomenon directly correlated with apoptotic events, we ran a semiquantitative analysis using confocal laser scanning microscopy. Results: Carbon quantum dots were synthesized and were successfully employed for Cyt c detection by means of fluorescence microscopy. Significant drops in fluorescence intensity were observed in the case of cells treated with apoptosis-inducing therapeutic compounds compared to untreated cells, confirming Cyt c release from mitochondria to cytosol. Conclusion: Considering these results, we strongly believe this method can contribute to an indirect in vitro evaluation of apoptosis.

## 1. Introduction

The total number of cells in an organism is rigorously regulated by physiological processes, which not only include cell division, but also a controlled rate of cell death. For a normal development and homeostasis, irreversibly damaged cells that become a threat to the body will either enter a senescent phase or undergo a form of programmed cell death. Apoptosis is a type of cell death characterized by the condensation of chromatin, fragmentation of nucleus, shrinkage of the cell and plasma membrane blebbing. The process involves the controlled activation of certain caspase proteases that rapidly degrade all cellular structures. Defective apoptosis is usually detrimental, being present in various diseases, including autoimmune diseases, neurodegenerative pathologies and cancers [1]. As a consequence of apoptosis involvement in these medical conditions, highlighting this process and discriminating it from other types of cell death such as necrosis and autophagy is a key step in cell-based screening of cytotoxic compounds [2].

Cytochrome c (Cyt c) is a hemeprotein associated with the inner mitochondrial membrane, which plays the role of an electron carrier necessary for ATP generation [3]. In normal conditions, the outer mitochondrial membrane is permeable to metabolites but not to proteins [4], making the translocation of Cyt c from the intermembrane space of the mitochondria into the cytoplasm an event specific to early apoptosis. This process is a key event in the intrinsic apoptosis pathway and is often defined as the irreversible point of undergoing apoptosis [2]. Since a small fraction of Cyt c can boost different amplification loops, which induce the release of the entire Cyt c from the mitochondria to the cytosol [4,5], a Cyt c concentration between 1 and 10 µM [2] can be detected. Cytosolic Cyt c induces the formation of the apoptosome (Apaf-1/caspase-9/cytochrome c complex) responsible for the degradation of cellular structures. As such, the quantification of Cyt c release is widely used to highlight mitochondria-dependent apoptosis [6], but the most common techniques are either time consuming or require complex procedures such as immunolabeling, subcellular fractionation and Western blotting [7].

Considering that optical imaging is a non-invasive and sensitive approach for the visualization of biological systems [8], fluorescent nanomaterials that are able to highlight cellular processes (including cellular death through apoptosis) by quantifying the concentration of specific molecules attracted significant attention in recent years [9,10,11]. This led to the development of a large diversity of fluorescent nanoparticle-based biosensors [12,13,14]. 

Carbon quantum dots (CQDs) represent a class of carbon-based fluorescent nanoparticles (NPs) that have drawn considerable attention, especially in the case of biomedical applications. Their unique optical and biocompatible properties and interaction with biomolecules brought them into the spotlight for modern biomedical applications. 

Most CQDs are quasi-spherical nanoparticles composed of an amorphous/crystalline core consisting of sp^2^ carbon atoms [15] and oxygen or nitrogen-based surface functional groups. They represent a new class of biocompatible, fluorescent nanomaterials exhibiting great stability, from the point of view of their morphology as well as of their luminescent properties [16,17]. They can be easily synthetized using cost-efficient procedures in aqueous media [18]. Furthermore, their size (on the order of a few nanometers) and large surface area, abundant in π-electrons, make them suitable for supramolecular binding of hydrophobic molecules through π-π interactions [19]. These attributes make them excellent candidates for drug delivery applications [20].

Most CQDs show an optical absorption spectrum in the UV region with a tail that extends to the visible domain [15]. Depending on the synthesis method [21] or surface modifications [22], their optical properties may be designed for a specific application [23,24]. 

In this paper, we synthesized a novel type of fluorescent, carbon-based nanoparticle, and used these nanoparticles as powerful optical sensors for the detection of Cyt c release from mitochondria to cytoplasm in order to experimentally prove the occurrence of the early apoptosis process. The as-synthesized CQDs showed remarkable physicochemical properties (fluorescence, high stability) in both neutral and acidic media in the presence of various biological molecules such as proteins and amino acids. They also exhibited a bright blue emission that specifically dropped in the presence of Cyt c. Based on this property, we conducted a semiquantitative analysis for the detection of cytosolic Cyt c using confocal laser scanning microscopy (CLSM).

## 2. Materials and Methods

### 2.1. Materials

Penicillin–streptomycin 100,000 UI/mL, poly(ethylenimine) (PEI) 1300~50% in H_2_O, sodium citrate, cytochrome c from equine heart, transferrin, fibrinogen, human serum albumin, bovine serum albumin, staurosporine from streptomyces, and 3-(4,5-dimethulthiazol-2-yl)-2,5-diphenyltetrazolium bromide (MTT) were purchased from Sigma-Aldrich, St. Louis, MO, USA. Slide-A-Lyzer^®^ Dialysis Cassette–2000 MWCO 3.0–12 mL Capacity and DRAQ5™ were purchased from Thermo Scientific, Rockford, IL, USA. l-Valine, l-Serine, l-Methionine, l-Leucine, l-Aspartic Acid, l-Tryptophan, l-Asparagine monohydrate, l-Phenylalanine were purchased from ThermoFischer (Kandel, Germany). Fetal bovine serum and phosphate saline buffer were purchased from Gibco, Grand Island, NY, USA. Etoposide was purchased from Fresenius Kabi, Bad Homburg, Germany. CytoPainter Mitochondrial Staining Kit Red Fluorescence was purchased from Abcam, Cambridge, UK. Phalloidin-FITC Acti-stainTM 488 Fluorescent Phalloidin was purchased from Cytoskeleton Inc., Denver, CO, USA. Prolong^®^ Gold Antifade Reagent Molecular Probes was purchased from Cell Signaling Technology, Danvers, CO, USA. Double distilled water (18.2 MΩ) was used as a solvent.

### 2.2. Carbon Quantum Dots Synthesis

CQDs were prepared approaching a bottom-up carbonization process using sodium citrate and PEI solutions as exclusive precursors. Briefly, 3 mL of PEI 1300 5% solution was mixed with 7 mL of 16 mM sodium citrate solution in a sealed Duran glass bottle and heated at 170 °C for 20 h. After this period, it was left to cool down at room temperature. Finally, 20 mL of Milli-Q water were added, and the resulted solution was sonicated for 20 min followed by centrifugation at 12,000× *g* for 10 min. The pale-yellow colored supernatant was transferred into a clean 25 mL glass bottle. The solution was then dialyzed against Milli-Q water over night in a 2 kDa MWCO (molecular weight cut-off) dialysis cassette and stored at 4 °C in a Duran glass bottle.

### 2.3. Optical Properties

#### 2.3.1. UV-Vis Spectroscopy

The absorbance, emission and excitation spectra of aqueous solutions were read using a Duetta™ Fluorescence and Absorbance Spectrometer (HORIBA Scientific, Kyoto, Japan). The absorbance spectrum was measured in the 250–800 nm wavelength interval using a step size of 1 nm. The fluorescence intensity was read in the 360–700 nm interval, with an emission wavelength step size of 0.5/1 nm. A 350 nm excitation wavelength (having a 5 nm bandwidth) was employed. For a proper evaluation of their optical properties, the excitation emission matrix (EEM) was constructed using HORIBA Scientific–EzSpec 1.3 software after analyzing the CQDs luminescent properties using different excitation wavelengths between 300 and 550 nm. The step increment of the excitation laser was set to 5 nm. In this case, the emitted light was collected in the 400–800 nm range using a bandpass of 5 nm.

For the calculation of the quantum yield (QY), we used quinine bisulphate as a standard [25,26]. Both CQDs and QBS solutions were kept at low concentrations, with an absorbance under 0.1 at 350 nm excitation to avoid inner filter effects and to assure a linear relationship between the molar concentration and absorbance [27]. 

Consequently, to test the stability of the as-synthesized CQDs, the fluorescence was measured using a 360 nm excitation and 440 nm emission in time intervals of 10 s for 1 h. Furthermore, the fluorescence emission intensity was measured at various pH values between 4 and 10. The CQDs were incubated for 1 h with different pH solutions and the fluorescence was measured using 360 nm excitation wavelength, emission range 370–800, Em/Ex bandpass 5 nm, integration time 0.05 s. 

Then, the CQDs stock solution was diluted in PBS 1X and mixed with various concentrations of Cyt c (0, 0.5, 1, 2.5, 5, 7.5, 10, 12.5, 15, 17.5, 20, 25, 50, 75, 100 µM). The emission spectrum of the solutions containing CQDs and Cyt c in different concentrations was recorded in the 370–800 nm range under 360 nm excitation light.

Another investigation focused on the CQDs fluorescence intensity variations as a result of Cyt c addition at different time points. Fluorescence spectra were measured every 10 s for 20 min. Cyt c was added up to a final concentration of 10, 20 and 30 µM, at 5, 10 and 15 min respectively. The fluorescence emission was detected between 370 and 600 nm. A 360 nm excitation wavelength was used, excitation and emission bandpasses were 5 nm, and the integration time was set to 0.05 s. 

The fluorescence quenching effect of CQDs solution, upon the addition of different biological molecules, was evaluated by UV–Vis absorbance (250–800 nm wavelength, step increment 1 nm, bandpass 5 nm, integration time 0.05 s) and emission (360 nm excitation wavelength, emission range 370–800, Em/Ex bandpass 5 nm, integration time 0.05 s) spectroscopy. CQDs optical properties were analyzed in the presence of various proteins (human serum albumin-HSA, bovine serum albumin-BSA, transferrin, fibrinogen and Cyt c) and amino acids (l-valine, l-aspartic acid, l-arginine, l-tryptophan, l-methionine, l-phenylalanine, l-leucine, l-serine) at 20 µM final concentration.

#### 2.3.2. Transmission Electron Microscopy

TEM was performed on a Hitachi HT7700 (Hitachi, Tokyo, Japan) equipped with an 8-megapixel XR81 camera (AMT, Woburn, MA, USA). A drop of the 10% diluted solution was drop-casted on a glow discharged carbon-coated copper grid and allowed to air dry completely. The sample was imaged at 80 kV in high-resolution mode using the 0.50 µm spot size. Images were processed for contrast based on the intensity histogram and calibrated in ImageJ 2.0.

#### 2.3.3. The Fourier Transform Infrared (FTIR)

FTIR spectra were recorded with a Bruker TENSOR II instrument (Bruker Optics Inc., Billerica, MA, USA) in attenuated total reflectance mode using the platinum attenuated total reflectance (ATR) accessory in the 400–4000 cm^−1^ spectral range with a resolution of 4 cm^−1^. The CQDs colloidal solution was allowed to dry on the diamond crystal, and the spectrum of 16 scans was recorded. The FTIR spectra of the precursors were recorded in a similar manner by placing the substance directly on the diamond crystal.

#### 2.3.4. Raman Spectroscopy

The Raman spectra were recorded using a Renishaw inVia Reflex Raman confocal multilaser spectrometer possessing a spectral resolution of 1 cm^−1^. The wavelength calibration was performed using an internal silicon reference. All Raman spectra were acquired using a 50× (NA = 0.75) Leica objective. A 785 nm diode laser (Renishaw, Gloucestershire, UK) was used for excitation. The laser power (measured at the sample surface) was ~63 mW. The exposure time was set to 1 s, and 20 accumulations were used for each measurement.

The spectrograph was equipped with a 600 lines/mm grating and a charge-coupled device camera (CCD). WiRE 4.2 software (Renishaw plc, Gloucestershire, UK) was used for data collection and spectral preprocessing, including cosmic ray removal and baseline correction. The latter was applied to all spectra to eliminate the fluorescence background. Carbon dots spectrum represents an average of 25 spectral acquisitions from different positions of the whole dried ring sample area.

### 2.4. Biological Characterization and In Vitro Studies

#### 2.4.1. Cell Culture

Adenocarcinoma human alveolar basal epithelial cells (A549) were purchased from LGC Standards GmbH (Wesel, Germany). The cells were maintained in Ham’s F12 medium (Sigma-Aldrich, St. Louis, MO, USA) supplemented with 10% FBS (Gibco, Grand Island, NY, USA), and 1% penicillin–streptomycin 10,000 UI/mL (Sigma-Aldrich, St. Louis, MO, USA) at 37 °C in a humidified atmosphere with 5% CO_2_.

#### 2.4.2. Cell Viability Assays

The A549 cells were grown at a density of 1 × 10^4^ cells/well in a 96-well plate. After 24 h, they were incubated with staurosporine (Sigma-Aldrich, St. Louis, MO, USA) at different concentrations (0.1, 1, 5, 10, 50, 100 µM) and respectively etoposide (10, 100, 250, 500, 750 nM, and 1, 2.5 µM) for an additional 24 h at 37 °C, 5% CO_2_, in a humidified incubator. The same steps were followed for the incubation of cells with CQDs. The absorbance at 570 nm, corresponding to the viability of the cells, was then measured using 3-(4,5-dimethulthiazol-2-yl)-2,5-diphenyltetrazolium bromide (MTT) assay (Sigma-Aldrich, St. Louis, MO, USA). The viability rate of the cells was calculated by applying two-way ANOVA and Bonferroni’s post hoc tests using Prism 5 software. The data were considered statistically significant at *p* < 0.05 and were illustrated as follows: *p* < 0.05, ** *p* < 0.01, *** *p* < 0.001 when compared with the untreated (control) group. The results were expressed as mean values ± SEM.

#### 2.4.3. Confocal Microscopy–Morphological Characterization

Cells were grown on chamber slides and treated with different concentrations of staurosporine or etoposide chosen in accordance with the calculated IC50 of these compounds for 24 h. After an incubation time of 4 h, the cells were fixed using 4% paraformaldehyde and permeabilized using 0.5% Triton X and stained with CytoPainter Mitochondrial Staining Kit–Red Fluorescence (Abcam, Cambridge, UK) for one hour, in fresh PBS, at 37 °C, under 5% CO_2_. Thereafter, the cells were washed three times in PBS buffer and fixed in 4% (*w*/*v*) paraformaldehyde for 10 min at room temperature. Phalloidin-FITC (Acti-stainTM 488 Fluorescent Phalloidin, Cytoskeleton Inc., Denver, CO, USA) was then added and incubated at room temperature for 30 min, in darkness. The cells were washed again three times with PBS and then the nuclei were stained with DAPI (Abcam, Cambridge, UK) 100 µg/mL for 30 s, followed by another step of washing with PBS. The chamber slides were mounted in Prolong™ Gold antifade mounting medium. Cells were imaged under an Olympus FLUOVIEW FV1200 laser scanning fluorescence confocal microscope. Image acquisition was performed using the UPLSAPO40 × 2 (0.95 NA) objective. The images were obtained using sequential mode (three channels: 405/488/543 nm excitation). Other settings for the image acquisition were determined depending on the fluorescent dyes by the software FV10-ASW 4.2. Images were processed using ImageJ. 

#### 2.4.4. Semiquantitative Analysis–Confocal

Cells were grown on chamber slides and incubated with 10% of the 1:10 solution of CQDs for 4 h. Thereafter, the cells were treated with 11.87 and 5.935 µM staurosporine or 195 and 97.5 µM etoposide for 4 h. The cells were then fixed using 4% PFA and the nuclei were labeled with DRAQ5™. The chamber slides were mounted as described above and examined using confocal laser scanning microscopy (CLSM). Image acquisition was performed with the UPLSAPO40 × 2 (0.95 NA) objective, using sequential mode (three channels: 405/633 nm excitation and one channel for differential interference contrast (DIC) microscopy). The acquisition parameters were left identical for all the samples in order to semi quantitatively compare the fluorescence intensity of the CQDs. Image analysis and processing were performed using ImageJ.

**Figure 1:**
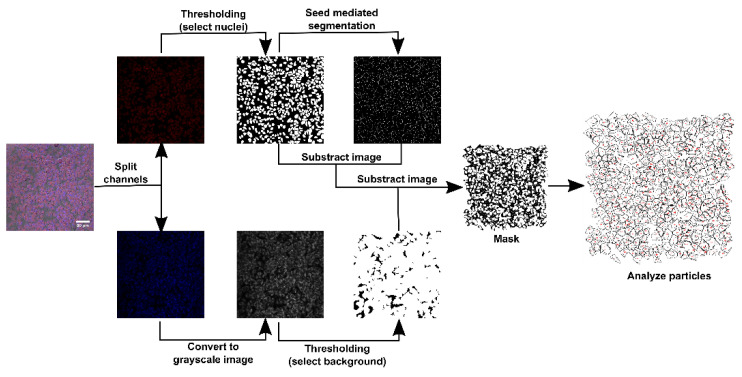
Pre-semiquantitative analysis image processing workflow.

After the image acquisition, we performed the semiquantitative analysis in ImageJ 2.0. The three channels (DIC, blue emission and red emission) were split. The channel corresponding to the blue light emission of the CQDs was transformed in a grayscale image. Thereafter, we applied a threshold filter in order to select pixels corresponding to the background noise. Consequently, we transformed this image into a 2-bit mask. The channel corresponding to the red-light emission of the DRAQ5 labeled nuclei was transformed in a 2-bit mask using a threshold filter. Thereafter, we ran a seed mediated segmentation starting from every nucleus. Then, we subtracted this image from the nuclei mask and the resulted image from the background mask. These steps (Figure 1) led to a mask of objects that correspond to cells without nuclei. This allowed us to calculate the mean intensity and area of every cell’s cytoplasm in the grayscale image corresponding to CQDs emission. This step was performed using the analyze objects function in ImageJ.

These steps were performed for 3 different images corresponding to every group (control, ETO25, ETO50, STAU25, STAU50). The intensity distribution was plotted for every group as compared to the control group, and different tests were performed to show the statistical significance.

We used a t test to verify if the means of the distributions were significantly different, assuming that the data was normally distributed (*p* < 1 − α if the means are different). Thereafter, we ran a Kolmogorov–Smirnov test to see whether the distributions were normal (*p* < 1 − α if the distribution is not normal) and a Mann–Whitney–Wilcoxon test to verify if the data sets came from the same distribution (*p* < 1 − α if the data sets come from different distributions).

## 3. Results and Discussion

### 3.1. Preparation and Characterization of CQDs

**Figure 2:**
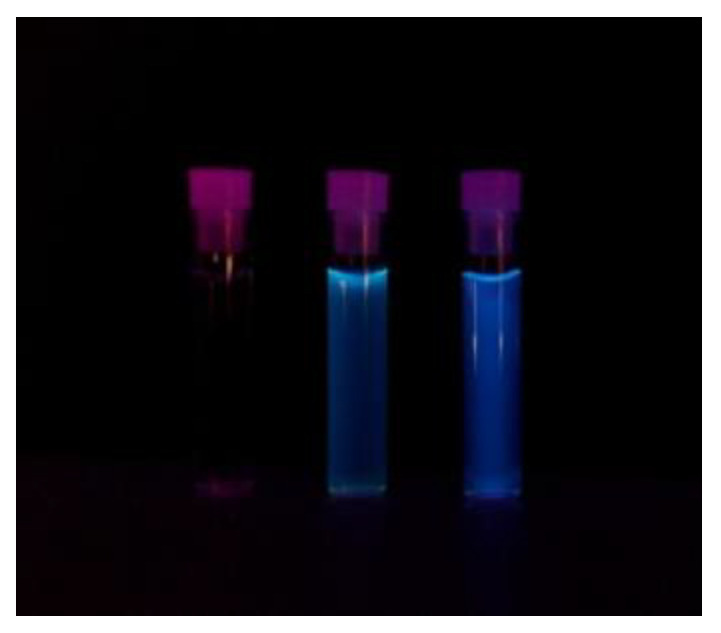
Optical images of three vials exposed to UV light: water (**left**), 10× diluted CQDs solution (**middle**) and non-diluted CQDs solution (**right**).

Over time, our research group has proposed original methods for the synthesis of different types of hybrid nanoparticles [28,29,30,31,32,33]. The CQDs colloidal solution was prepared, as previously described in the Methods section, based on a novel synthesis protocol developed in our laboratory using, as precursor materials only, PEI and sodium citrate. Thereafter, dialysis of the yellow solution was conducted at room temperature against distilled water for 24 h. Bright blue fluorescence emission light was observed when the solution was exposed to UV light (Figure 2), thus confirming the presence of fluorescent CQDs.

### 3.2. Optical Properties

**Figure 3:**
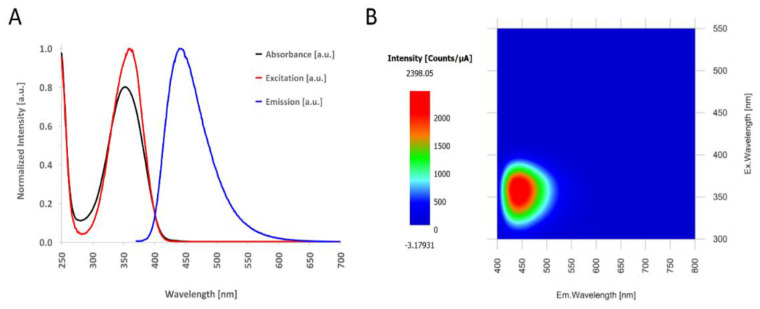
(**A**) The absorbance spectrum of the as-prepared CQDs solution (black), the excitation spectrum (red) and the emission spectrum of the CQDs (blue) when exposed to a 360 nm excitation light. (**B**) Excitation and emission matrix (EEM) of the as-prepared CQD solution.

The optical properties of the synthesized CQDs were first characterized by UV–Vis measurements. Absorbance and emission spectra were collected. The as-prepared CQDs presented high absorbance in the UV region with a peak located at 353 nm and a tail extending to the visible spectral domain. The far broad UV peak is attributed to the π-π* transition of the aromatic sp^2^ structure, while the peak around 353 nm represents a molecular band as a result of n-π* transitions of structures containing N or O (Figure 3A) [34]. 

The CQDs aqueous solution presented blue fluorescence emission under UV illumination. The maximum emission intensity was detected at 440 nm, under 360 nm excitation wavelength, as can be seen in Figure 3.

The quantum yield (QY) of the as-synthesized CQDs was measured using QBS in 0.1 M H_2_SO_4_ as reference (QY = 0.54). Both CQDs and standard solutions were kept at low concentrations, with an absorbance under 0.1 at 360 nm excitation to avoid inner filter effects and to assure a linear relationship between the molar concentration and absorbance. The absorbance at 360 nm was recorded in triplicate, and the QY of the CQDs was calculated using the procedure proposed by Demas and Crosby [27], using the following equation:

$$\varphi_{X}=\varphi_{\mathrm{st}}\;\left(\frac{A_{X}}{A_{\mathrm{st}}}\right)\left(\frac{{\left(\mathrm{Abs}\right)}_{\mathrm{st}}}{{\left(\mathrm{Abs}\right)}_{X}}\right){\left(\frac{\eta_{X}}{\eta_{\mathrm{st}}}\right)}^{2}$$

where φ is the QY, A is the area under the fluorescence curve, Abs is the absorbance at 360 nm and η is the refractive index of the solvent. The subscripts “x” and “st” stand for sample and standard. 

The refractive index of both solvents: water for CQDs solution and H_2_SO_4_ aqueous solution for the standard sample is 1.33. The calculated fluorescence QY for the as-prepared CQDs is 11.3%.

**Figure 4:**
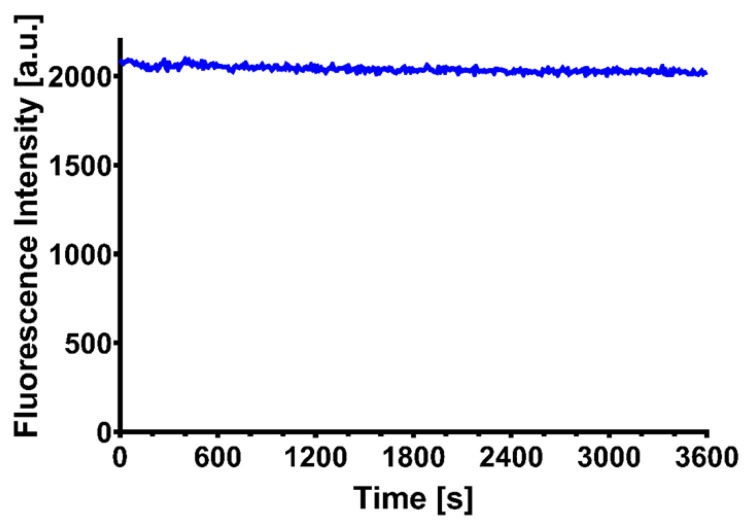
Assessment of CQDs fluorescence intensity under continuous irradiation for 1 h.

The fluorescence intensity of CQDs was measured for 1 h to evaluate their stability, using a 360 nm excitation wavelength. No significant decrease of fluorescence intensity was detected, as can be seen in Figure 4.

**Figure 5:**
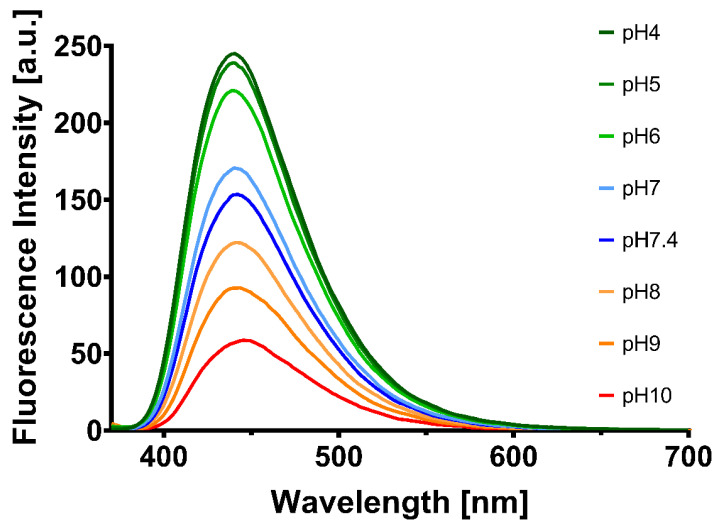
The stability of CQDs solutions in PBS at various pH values.

Moreover, the stability of the NPs was investigated at different pH values, as shown in Figure 5. The CQDs showed high stability in acidic media (high fluorescence intensity at pH 4–7), while their intensity decreased significantly at higher pH values.

**Figure 6:**
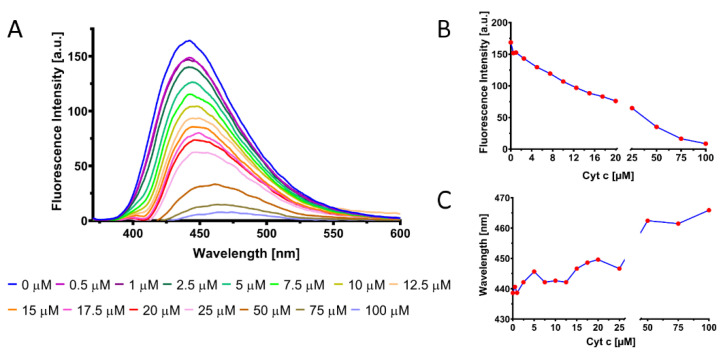
(**A**) Spectra showing the fluorescence intensity of CQDs in the presence of various concentrations of Cyt c. (**B**) Relationship between Cyt c concentration and CQDs maximum fluorescence intensity. (**C**) Lambda shift exhibited by the CQDs solution in the presence of various Cyt c concentrations.

The correlation between the fluorescence intensity of CQDs and the concentration of Cyt c is highlighted in Figure 6. The photoluminescence intensity of the as-synthesized CQDs decreases in the presence of Cyt c in a concentration dependent manner (Figure 6A). Cyt c acts as a quencher for these nanoparticles, probably through a mechanism called inner filter effect (IFE), which involves the emitted light reabsorption by Cyt c. The overlap between the absorption spectrum of Cyt c and the emission spectrum of the CQDs supports this statement as shown in Supplementary Figure S1. These differences in the emission intensity (Figure 6B) of the cell internalized CQDs in the presence of Cyt c could be detected in in vitro studies, making thus possible the distinction between apoptotic cells and nonapoptotic ones. The same concentration of CQDs was mixed with different concentrations of Cyt c (0, 0.5, 1, 2.5, 5, 7.5, 10, 12.5, 15, 17.5, 20, 25, 50, 75, 100 µM) and the fluorescence intensity under 360 nm excitation was measured. A small lambda shift (~30 nm), proportional with Cyt c concentration, was recorded as can be observed in Figure 6C.

**Figure 7:**
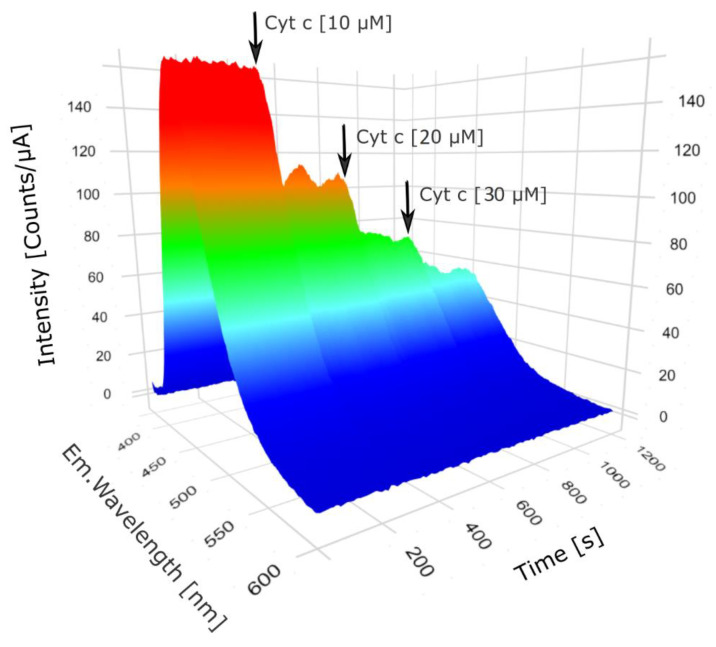
CQDs fluorescence intensity spectra variation in 20 min at different doses of Cyt c. Starting with 5 min time point, Cyt c was added, and the effects on fluorescence intensity were investigated for 5 min before increasing the dose.

The quenching effect of Cyt c on fluorescence intensity of CQDs was assessed in time. CQDs fluorescence was measured for 20 min at 3 time points by varying Cyt c concentration from 0 to 10, 20 and 30 µM (Figure 7). An indirectly proportional relation between Cyt c concentration and CQDs fluorescence intensity was observed, meaning that at high Cyt c concentrations, the fluorescence intensity of these nanoparticles is decreased.

**Figure 8:**
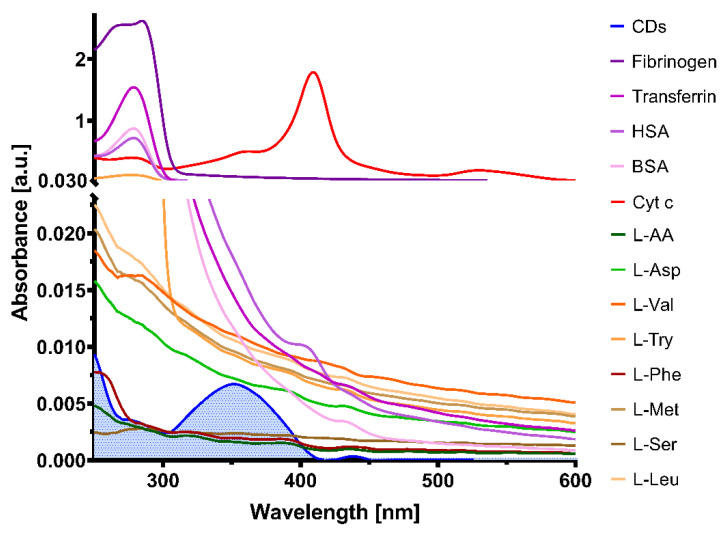
Absorbance spectra of different biological molecules that could interfere with the CQDs optical properties. The absorbance of CQD solution is highlighted in blue.

**Figure 9:**
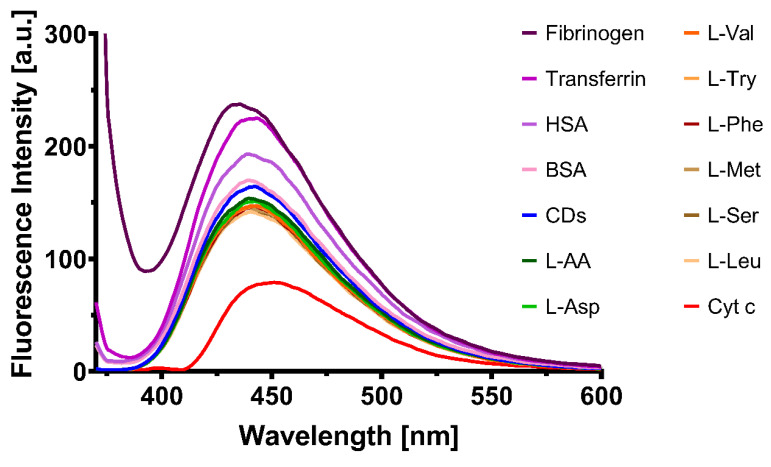
Emission spectra of CQDs in the absence and presence of biological molecules.

To test the hypothesis that the quenching effect is obtained mostly due to the presence of Cyt c in the proximity of CQDs, other biological molecules such as human serum albumin, bovine serum albumin, fibrinogen, transferrin and amino acids were investigated. In this regard, absorbance and emission spectra were collected to highlight possible interferences of these molecules at 20 µM with CQDs spectra. Absorbance spectra was read between 250–800 nm (Figure 8), while emission spectra were read between 370–800 nm with excitation at 360 nm (Figure 9). 

A relationship between the Cyt c absorbance wavelength range and the CQDs emission spectra was identified, which could explain the quenching of the CQDs in the presence of this molecule. The other tested molecules showed no significant relation with the CQDs optical properties; therefore, the fluorescence intensity of the CQDs in the presence of these biological compounds was unlikely to happen. Moreover, another heme protein (hemoglobin, HGB) similar to Cyt c was tested to investigate if the structure is responsible for the quenching mechanism of CQDs. As shown in Supplementary Figure S2, HGB did not affect the fluorescence emission of CQDs; although, HGB presented an absorption peak between 400 and 600 nm (Supplementary Figure S3). These data prove that the quenching was due to Cyt c specifically and not to all heme proteins. 

### 3.3. Transmission Electron Microscopy (TEM)

**Figure 10:**
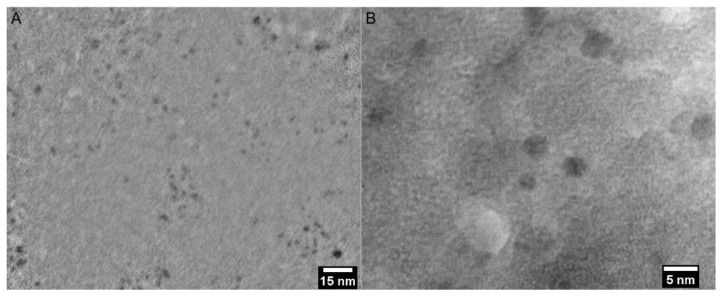
TEM image of the as-synthesized CQDs. 200k magnification image of the as-prepared CQDs (**A**) and ×600 k magnification image (**B**).

TEM was used to estimate the size, morphology, and dispersity of the as-synthesized CQDs. The nanoparticles were monodispersed, had a spherical morphology and a uniform size, ranging between 1 and 3 nm (Figure 10). No noticeable aggregates were found throughout the sample.

### 3.4. Fourier Transform Infra-Red Spectroscopy (FTIR)

**Figure 11:**
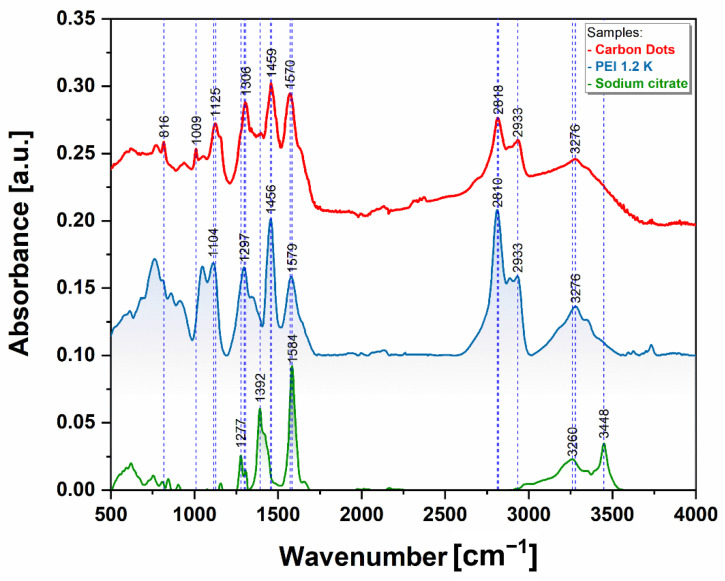
FTIR spectra of the CQDs (red) and of the precursors, sodium citrate (green) and PEI 1.2 K (blue) in the spectral range 400–4000 cm^−1^. The spectra were baseline corrected, normalized, and vertically shifted for better clarity.

The FTIR spectrum of the CQDs presents many similarities with the compounds used in their synthesis, especially PEI. As can be seen in Figure 11, at high wavenumbers, the main vibrations are at 2815 and 2932 cm^−1^ which correspond to the CH symmetric and asymmetric stretching vibration and the 3276 cm^−1^ is assigned to the NH stretching vibration [35]. In the fingerprint region, the peaks at 1570, 1458 and 1305 cm^−1^ may be assigned to the N–H bending, C–H bending and C–N stretching vibrations, respectively [36]. 

### 3.5. Raman Spectroscopy

**Figure 12:**
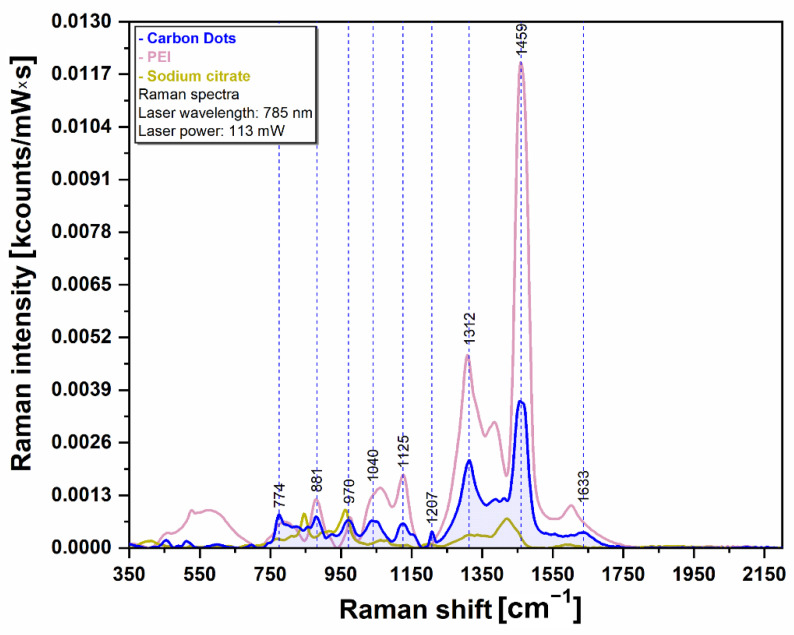
Raman spectra of the CQDs (blue) and of the precursors sodium citrate (light brown) and PEI 1.2 K (light pink) in the spectral range 350–2150 cm^−1^. All the spectra were baseline corrected for better clarity.

Raman spectroscopy was employed in order to further prove the molecular species present on the outer surface of the CQDs (Figure 12). As such, we recorded the spectrum of the CQDs (blue curve) together with the spectra of the only two precursors that were used during the synthesis: PEI (magenta curve) and sodium citrate (olive curve). The spectra were recorded using a NIR excitation laser (785 nm). The Raman spectrum of the CQDs presents many similarities with the only two compounds employed in their synthesis. The major vibrational peaks detected in the case of CQDs (881, 970, 1040, 1125, 1312 and 1459 cm^−1^) can be assigned to different vibrational modes of PEI. The presence of these PEI specific vibrational bands represents irrefutable proof of the presence of this biocompatible polymer on the outer surface of CQDs as it was also shown by means of FTIR spectroscopy.

### 3.6. In Vitro Cytotoxicity Assessments of the CQDs Solution 

**Figure 13:**
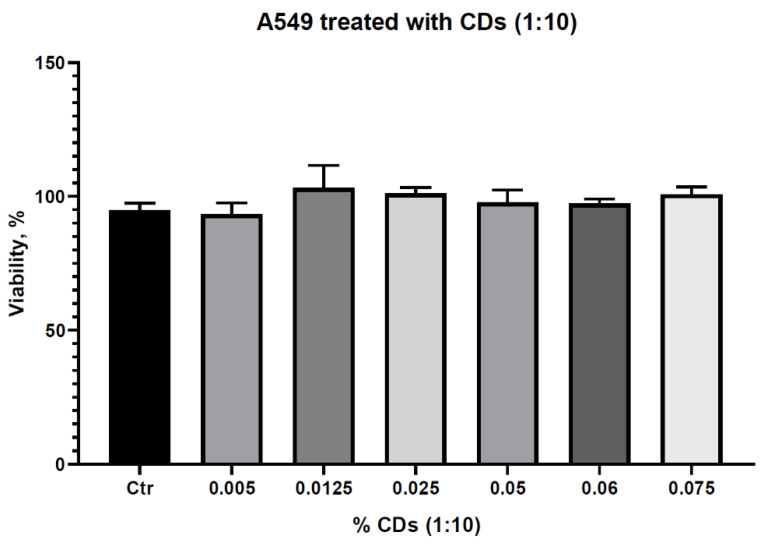
Viability rate of A549 cells toward various concentrations of the as-prepared CQDs solution. Based on the 24 h MTT assay, the viability rate was comparatively plotted between cells treated with different concentrations of CQDs solutions and untreated cells (control group). No statistically significant cytotoxicity of the nanoparticles was highlighted.

As estimated from previous literature studies, the CQDs solution showed negligible cytotoxicity [37] on A549 cells proven by an MTT assay for different concentrations of the as-prepared colloidal solution (0.075%, 0.06%, 0.05%, 0.025%, 0.0125%, 0.005%) (Figure 13). All the viabilities were higher than 90%, suggesting that the CQDs were not cytotoxic.

**Figure 14:**
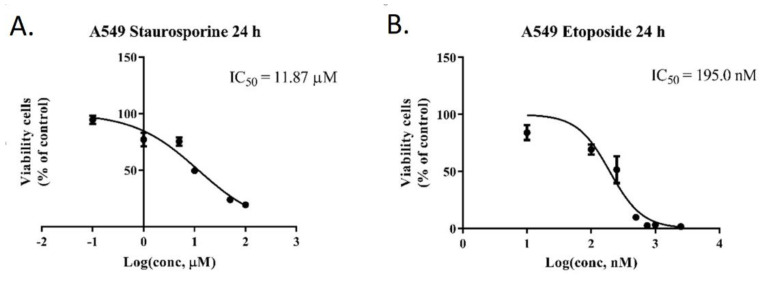
Viability rate of A549 cells treated with various concentrations of staurosporine (**A**) and etoposide (**B**).

A549 cells were incubated with different concentrations of staurosporine (0.1, 1, 5, 10, 50, 100 µM) and Etoposide (10, 100, 250, 500, 750 nM, and 1, 2.5 µM) for 24 h. The viability rate was then calculated, and a two-way ANOVA test indicated the statistical significance between the viability of the treated cells and the control group (Figure 14). As expected, a higher concentration led to a higher rate of cellular death. Based on the viability rate, the IC50 of 11,87 µM for staurosporine and 195 nM for etoposide were determined for A549 cells. 

Based on the 24 h MTT assay, the viability rate of A549 cells treated with staurosporine and etoposide at different concentrations was plotted and compared with untreated cells (control group). Higher concentrations of the two apoptosis inducing compounds affected the A549 cell line viability rate in a more significant manner. Based on these results, we were able to calculate an IC50 of 11.87 µM for staurosporine and an IC50 of 195 nM for etoposide.

### 3.7. Cellular Uptake

**Figure 15:**
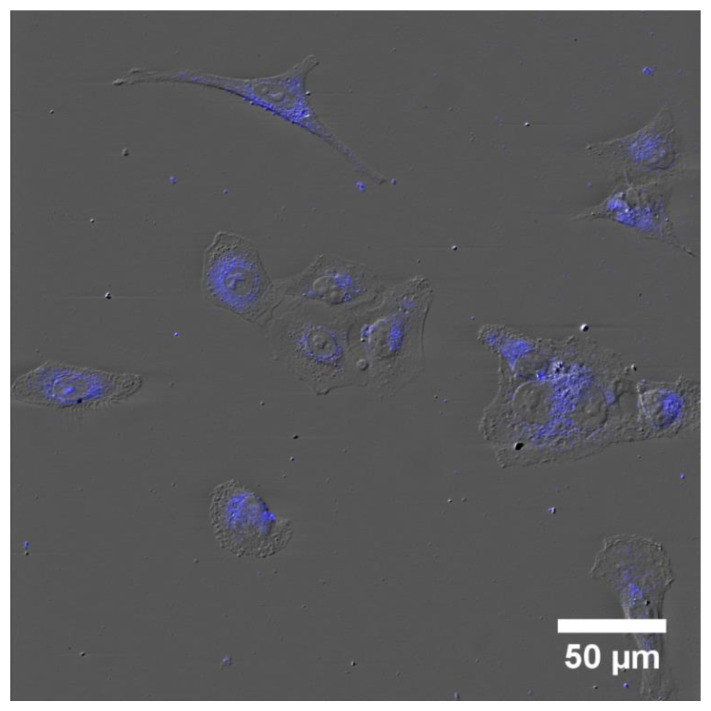
Cellular internalization of CQDs in A549 cells.

A549 cells were grown in chamber slides and treated with a 10% solution of the as-prepared CQDs for 4 h and mounted in Prolong Gold Mounting Medium for confocal microscopy observations. The specimen was excited using a 405 nm laser and scanned using two channel mode (DIC and blue emission). The DIC images clearly presented the cells and their nuclei, and the blue fluorescence confirmed the internalization of the CQDs (Figure 15). We observed low fluorescence intensity in the nucleus, suggesting that only a small amount of fractions of the carbon dots could pass the nuclear envelope, probably due to their physicochemical properties and not their size. The surface chemistry of the NPs is a key factor in cellular and subcellular level internalization [38]. Conversely, it was reported that metallic NPs smaller than 9 nm could freely bypass the nuclear envelope [39].

### 3.8. Confocal Microscopy—Morphological Characterization

Morphological changes induced by staurosporine or etoposide in A549 cells reveals nuclei and cytoskeleton damages. Staurosporine is known as an apoptosis-inducing drug [40] that leads to actin and tubulin reorganization [41,42], chromatin coagulation and mitochondria swelling [43].

**Figure 16:**
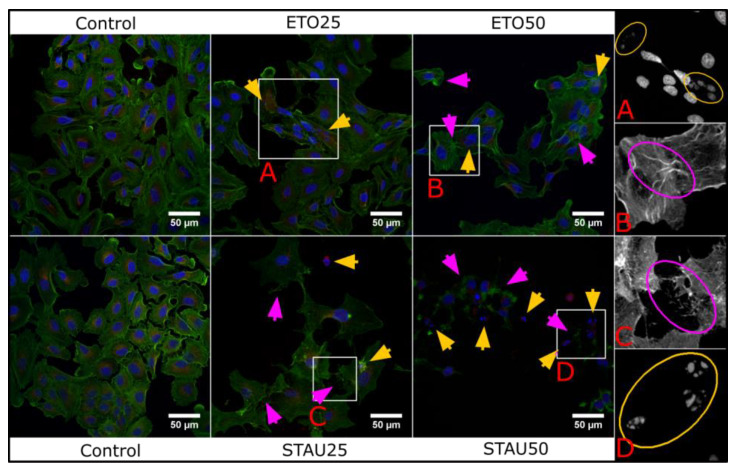
Morphological changes induced by Eto and Stau in A549 cell line at IC25 and IC50. Yellow arrows indicate nuclear fragmentation/damage and magenta arrows show cytoskeleton degradation. The panel in the right presents single channel regions of interest to highlight the structural features in a more detailed manner.

Etoposide is a candidate for DNA topoisomerase II inhibition and acts in the late S and G2 phases of cell cycle [44], determining nuclei swelling [45] and activating apoptosis [46]. We tested these two apoptotic compounds on A549 cell cultures to evaluate the morphological changes at IC50 and IC25 doses (Figure 16). Similar to literature studies, we observed cytoskeleton and nuclei fragmentation.

### 3.9. Semiquantitative Analysis

**Figure 17:**
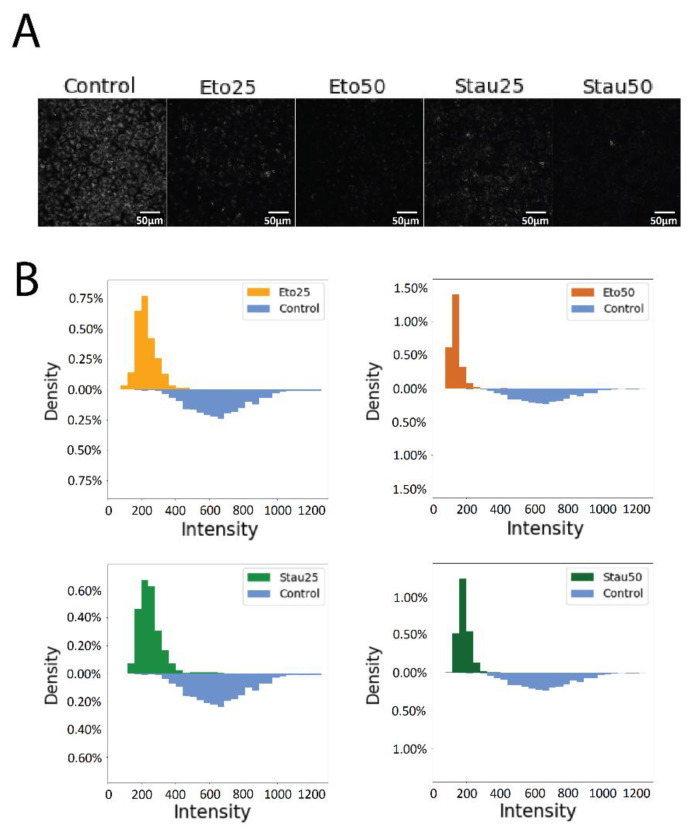
Semiquantitative analysis of the cells treated with Eto and Stau. Grayscale confocal microscopy images of internalized CQDs in A549 cells treated with IC25 and IC50 of ETO and Stau (**A**) and graphical representation of CQDs fluorescence intensity variation of treated cells compared to the untreated cells (**B**).

A semiquantitative analysis of the grayscale images corresponding to the blue fluorescence of the CQDs internalized in A549 cells treated with these two candidate drugs (Figure 17A), the plotted intensity of the cells in the control group, and the intensities of all the cells belonging in the four experimental groups compared to the control group are presented in Figure 17B. A drop in the fluorescence intensity can be clearly seen in the images of the treated cells as compared to the control group.

**Figure 18:**
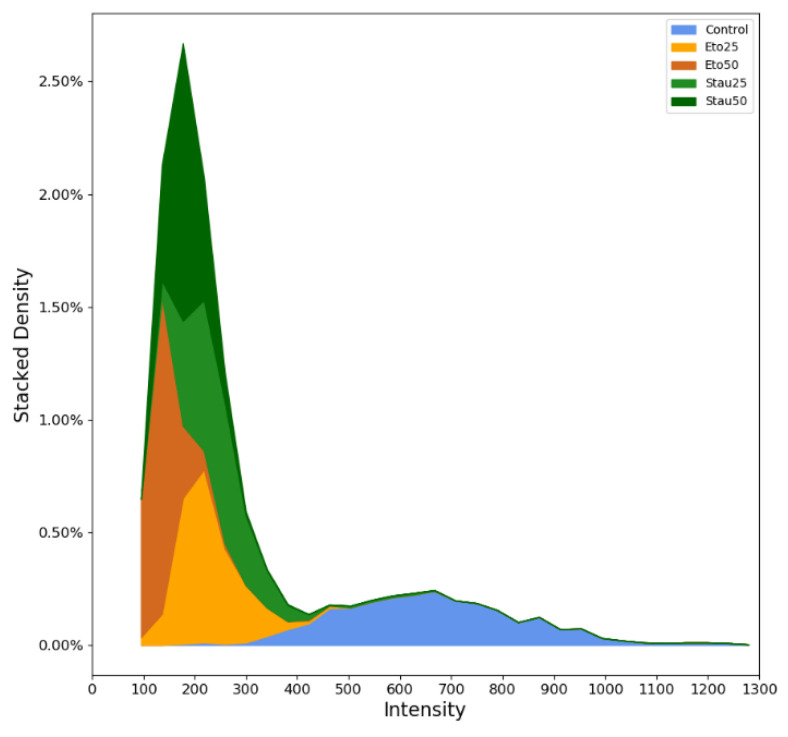
Stacked plot for the semiquantitative analysis showing the distribution of cells based on their fluorescence intensity.

The differences in fluorescence intensity between the groups treated with the proapoptotic compounds and the control group correspond to the quenching capacity of Cyt c on the as-prepared CQDs. They are illustrated for every group as compared to the control in a stacked plot (Figure 18). Both IC50 and IC25 for Stau and Eto induced mitochondrial permeabilization followed by Cyt c release. We highlighted this through the quenching of the CQDs, which is indirectly proportional to the drop in the mean intensity.

The statistical significance of the mean intensity of the groups corresponding to the treated cells compared to the control group are illustrated in Table 1 as *p* values for three different tests (*t* test, Kolmogorov-Smirnov and Mann-Whitney-Wilcoxon). All the groups presented statistically significant changes in the mean intensity (*p* < 0.0001).

## 4. Conclusions

We performed a semiquantitative analysis of cytosolic concentration under induced apoptosis conditions from cells using blue fluorescent CQDs. These CQDs presented remarkable qualitative physicochemical properties as optical features, high stability in both neutral and acidic media, but also in the presence of various biological molecules such as proteins and amino acids. Moreover, CQDs fluorescence intensity was measured in time in different conditions and proved no significant dropping in it when exposed to 360 nm excitation wavelength.

## Figures and Tables

**Table 1:** Statistical analysis.

Group	*t* Test	Wilcoxon-Mann-Whitney	Kolmogorov-Smirnov
Ctrl	1	0.999962061	0.063092048
Eto25	4.47 × 10^−283^	2.80 × 10^−183^	0.009255334
Eto50	0	1.58 × 10^−193^	4.92 × 10^−5^
Stau25	0	8.51 × 10^−225^	4.18 × 10^−5^
Stau50	0	3.49 × 10^−220^	0.003144836

PDI = polydispersity index.

## Data Availability

Data is contained within the article or supplementary material.

## Supplementary Materials

The following are available online at https://www.mdpi.com/article/10.3390/pharmaceutics13101556/s1, Figure S1: Normalized absorbance and emission spectra of Cyt c (20 µM) and CQDs solutions, Figure S2: Emission spectra of pure CQDs (blue curve) and of CQDs in the presence of 20 µM hemoglobin (grey curve), Figure S3: Absorbance spectrum of hemoglobin.

## References

[1] Tait S.W.G., Green D.R. (2010). Mitochondria and cell death: Outer membrane permeabilization and beyond. Nat. Rev. Mol. Cell Biol..

[2] Zhang H., Zhang B., Di C., Ali M.C., Chen J., Li Z., Si J., Zhang H., Qiu H. (2018). Label-free fluorescence imaging of cytochrome c in living systems and anti-cancer drug screening with nitrogen doped carbon quantum dots. Nanoscale.

[3] Chandel N.S. (2014). Mitochondria as signaling organelles. BMC Biol..

[4] Kroemer G., Galluzzi L., Brenner C. (2007). Mitochondrial Membrane Permeabilization in Cell Death. Physiol. Rev..

[5] Garrido C., Galluzzi L., Brunet M., Puig P.E., Didelot C., Kroemer G. (2006). Mechanisms of cytochrome c release from mitochondria. Cell Death Differ..

[6] Campos C.B.L., Paim B.A., Cosso R.G., Castilho R.F., Rottenberg H., Vercesi A.E. (2006). Method for monitoring of mitochondrial cytochrome c release during cell death: Immunodetection of cytochrome c by flow cytometry after selective permeabilization of the plasma membrane. Cytom. Part A.

[7] Waterhouse N.J., Trapani J.A. (2003). A new quantitative assay for cytochrome c release in apoptotic cells. Cell Death Differ..

[8] Amin R.M., Elfeky S.A., Verwanger T., Krammer B. (2017). Fluorescence-based CdTe nanosensor for sensitive detection of cytochrome C. Biosens. Bioelectron..

[9] Jin M., Liu X., Zhang X., Wang L., Bing T., Zhang N., Zhang Y., Shangguan D. (2018). Thiazole Orange-Modified Carbon Dots for Ratiometric Fluorescence Detection of G-Quadruplex and Double-Stranded DNA. ACS Appl. Mater. Interfaces.

[10] Wang J., Qiu J. (2016). A review of carbon dots in biological applications. J. Mater. Sci..

[11] Das P., Maity P.P., Ganguly S., Ghosh S., Baral J., Bose M., Choudhary S., Gangopadhyay S., Dhara S., Das A.K. (2019). Biocompatible carbon dots derived from κ-carrageenan and phenyl boronic acid for dual modality sensing platform of sugar and its anti-diabetic drug release behavior. Int. J. Biol. Macromol..

[12] Sharma A., Khan R., Catanante G., Sherazi T., Bhand S., Hayat A., Marty J. (2018). Designed Strategies for Fluorescence-Based Biosensors for the Detection of Mycotoxins. Toxins.

[13] Yoo D., Park Y., Cheon B., Park M.-H. (2019). Carbon Dots as an Effective Fluorescent Sensing Platform for Metal Ion Detection. Nanoscale Res. Lett..

[14] Zarei-Ghobadi M., Mozhgani S.-H., Dashtestani F., Yadegari A., Hakimian F., Norouzi M., Ghourchian H. (2018). A genosensor for detection of HTLV-I based on photoluminescence quenching of fluorescent carbon dots in presence of iron magnetic nanoparticle-capped Au. Sci. Rep..

[15] Wang R., Lu K.-Q., Tang Z.-R., Xu Y.-J. (2017). Recent progress in carbon quantum dots: Synthesis, properties and applications in photocatalysis. J. Mater. Chem. A.

[16] Wang Y., Zhu Y., Yu S., Jiang C. (2017). Fluorescent carbon dots: Rational synthesis, tunable optical properties and analytical applications. RSC Adv..

[17] Dager A., Uchida T., Maekawa T., Tachibana M. (2019). Synthesis and characterization of Mono-disperse Carbon Quantum Dots from Fennel Seeds: Photoluminescence analysis using Machine Learning. Sci. Rep..

[18] Sun X., Lei Y. (2017). Fluorescent carbon dots and their sensing applications. TrAC Trends Anal. Chem..

[19] Hong G., Diao S., Antaris A.L., Dai H. (2015). Carbon Nanomaterials for Biological Imaging and Nanomedicinal Therapy. Chem. Rev..

[20] Feng T., Zhao Y. (2019). Preparation of Responsive Carbon Dots for Anticancer Drug Delivery. Methods Mol. Biol..

[21] Sutanto H., Alkian I., Romanda N., Lewa I.W.L., Marhaendrajaya I., Triadyaksa P. (2020). High green-emission carbon dots and its optical properties: Microwave power effect. AIP Adv..

[22] Li X., Zhang S., Kulinich S.A., Liu Y., Zeng H. (2015). Engineering surface states of carbon dots to achieve controllable luminescence for solid-luminescent composites and sensitive Be^2+^ detection. Sci. Rep..

[23] Zhou Y., Mintz K.J., Sharma S.K., Leblanc R.M. (2019). Carbon Dots: Diverse Preparation, Application, and Perspective in Surface Chemistry. Langmuir.

[24] Ali H., Ghosh S., Jana N.R. (2020). Fluorescent carbon dots as intracellular imaging probes. WIREs Nanomed. Nanobiotechnol..

[25] Fletcher A.N. (1969). Quinine Sulfate as a Fluorescence Quantum Yield Standard. Photochem. Photobiol..

[26] Eastman J.W. (1967). Quantitative Spectrofluorimetry-The Fluorescence Quantum Yield of Quinine Sulfate. Photochem. Photobiol..

[27] Crosby G.A., Demas J.N. (1971). Measurement of photoluminescence quantum yields. Review. J. Phys. Chem..

[28] Nițică Ș., Moldovan A.I., Toma V., Moldovan C.S., Berindan-Neagoe I., Știufiuc G., Lucaciu C.M., Știufiuc R. (2018). PEGylated Gold Nanoparticles with Interesting Plasmonic Properties Synthesized Using an Original, Rapid, and Easy-to-Implement Procedure. J. Nanomater..

[29] Știufiuc G., Toma V., Moldovan A., Știufiuc R. (2017). One Pot Microwave Assisted Synthesis of Cyclodextrins Capped Spherical Gold Nanoparticles. Dig. J. Nanomater. Biostruct..

[30] Stiufiuc R., Iacovita C., Nicoara R., Stiufiuc G., Florea A., Achim M., Lucaciu C.M. (2013). One-Step Synthesis of PEGylated Gold Nanoparticles with Tunable Surface Charge. J. Nanomater..

[31] Stiufiuc R., Iacovita C., Stiufiuc G., Florea A., Achim M., Lucaciu C.M. (2015). A new class of pegylated plasmonic liposomes: Synthesis and characterization. J. Colloid Interface Sci..

[32] Știufiuc G.F., Nițică Ș., Toma V., Iacoviță C., Zahn D., Tetean R., Burzo E., Lucaciu C.M., Știufiuc R.I. (2019). Synergistical Use of Electrostatic and Hydrophobic Interactions for the Synthesis of a New Class of Multifunctional Nanohybrids: Plasmonic Magneto-Liposomes. Nanomaterials.

[33] Iacovita C., Florea A., Scorus L., Pall E., Dudric R., Moldovan A.I., Stiufiuc R., Tetean R., Lucaciu C.M. (2019). Hyperthermia, Cytotoxicity, and Cellular Uptake Properties of Manganese and Zinc Ferrite Magnetic Nanoparticles Synthesized by a Polyol-Mediated Process. Nanomaterials.

[34] Carbonaro C.M., Corpino R., Salis M., Mocci F., Thakkar S.V., Olla C., Ricci P.C. (2019). On the Emission Properties of Carbon Dots: Reviewing Data and Discussing Models. C J. Carbon Res..

[35] Papavlu A.P., Dinca V., Filipescu M., Dinescu M. (2017). Matrix-Assisted Pulsed Laser Evaporation of Organic Thin Films: Applications in Biology and Chemical Sensors. Laser Ablation—From Fundamentals to Applications.

[36] Ţucureanu V., Matei A., Avram A.M. (2016). FTIR Spectroscopy for Carbon Family Study. Crit. Rev. Anal. Chem..

[37] Pelaz B., Alexiou C., Alvarez-Puebla R.A., Alves F., Andrews A.M., Ashraf S., Balogh L.P., Ballerini L., Bestetti A., Brendel C. (2017). Diverse Applications of Nanomedicine. ACS Nano.

[38] Jung Y.K., Shin E., Kim B.-S. (2015). Cell Nucleus-Targeting Zwitterionic Carbon Dots. Sci. Rep..

[39] Pan L., Liu J., Shi J. (2018). Cancer cell nucleus-targeting nanocomposites for advanced tumor therapeutics. Chem. Soc. Rev..

[40] Belmokhtar C.A., Hillion J., Ségal-Bendirdjian E. (2001). Staurosporine induces apoptosis through both caspase-dependent and caspase-independent mechanisms. Oncogene.

[41] Olguín-Albuerne M., Domínguez G., Morán J. (2014). Effect of Staurosporine in the Morphology and Viability of Cerebellar Astrocytes: Role of Reactive Oxygen Species and NADPH Oxidase. Oxid. Med. Cell. Longev..

[42] Nishi K., Schnier J., Brandbury M. (2002). Cell Shape Change Precedes Staurosporine-Induced Stabilization and Accumulation of p27kip1. Exp. Cell Res..

[43] Wang Y., Yang H., Liu H., Huang J., Song X. (2009). Effect of staurosporine on the mobility and invasiveness of lung adenocarcinoma A549 cells: An in vitro study. BMC Cancer.

[44] Montecucco A., Zanetta F., Biamonti G. (2015). Molecular mechanisms of etoposide. EXCLI J..

[45] Shin H.-J., Kwon H.-K., Lee J.-H., Anwar M.A., Choi S. (2016). Etoposide induced cytotoxicity mediated by ROS and ERK in human kidney proximal tubule cells. Sci. Rep..

[46] Sverchinsky D., Nikotina A., Komarova E., Mikhaylova E., Aksenov N., Lazarev V., Mitkevich V., Suezov R., Druzhilovskiy D., Poroikov V. (2018). Etoposide-Induced Apoptosis in Cancer Cells Can Be Reinforced by an Uncoupled Link between Hsp70 and Caspase-3. Int. J. Mol. Sci..

