# Supplementary material for: Quantifying Cytosolic Cytochrome c Concentration Using Carbon Quantum Dots as a Powerful Method for Apoptosis Detection
Source: Pharmaceutics. 2021 Sep 25;13(10):1556. doi: 10.3390/pharmaceutics13101556 (PMC8537359; doi:10.3390/pharmaceutics13101556)
Supplement: Supplementary file 1 [file pharmaceutics-13-01556-s001.zip › pharmaceutics-1350494-supplementary.pdf]

pharmaceutics

MDPI

Supplementary Materials: Quantifying Cytosolic Cytochrome c Concentration Using Carbon Quantum Dots as a Powerful Method for Apoptosis Detection

Cristian Silviu Moldovan, Anca Onaciu, Valentin Toma, Radu Marginean, Alin Moldovan, Adrian Bogdan Tigu, Gabriela Fabiola Stiufiuc, Constantin Mihai Lucaciu and Rares Ionut Stiufiuc

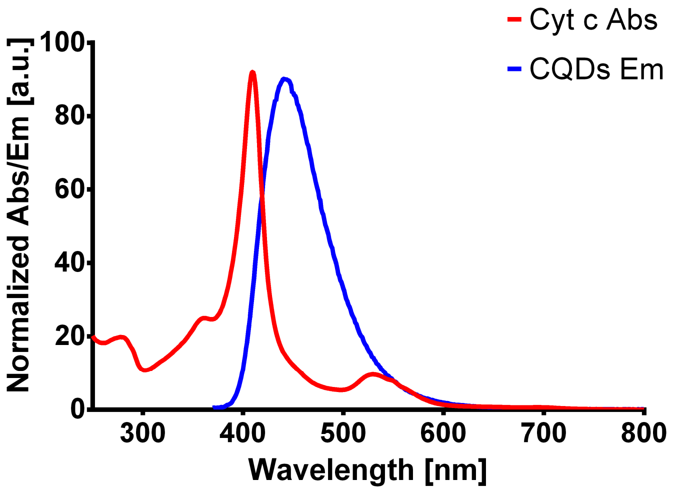

Figure S1. Normalized absorbance and emission spectra of Cyt c (20µM) and CQDs solutions.

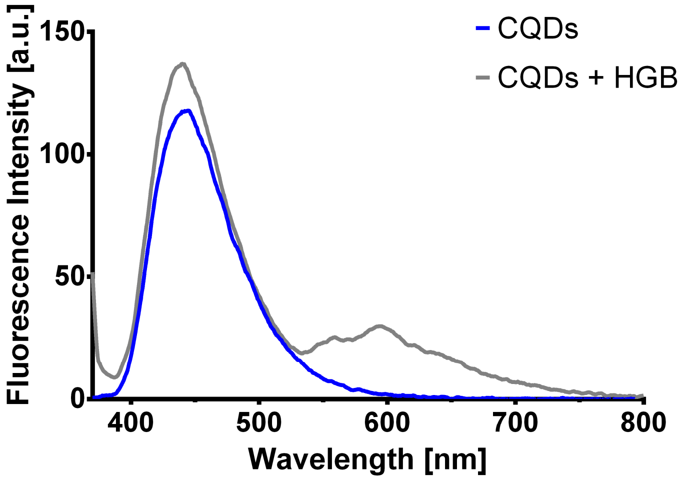

Figure S2. Emission spectra of pure CQDs (blue curve) and of CQDs in the presence of 20 µM haemoglobin (grey curve).

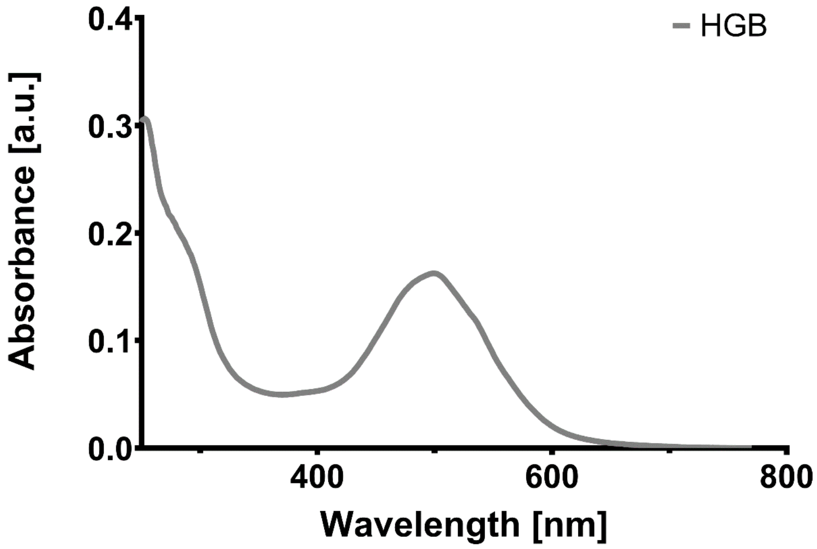

Figure S3. Absorbance spectrum of haemoglobin.
